# Fifty years of the mitochondrial pyruvate carrier: New insights into its structure, function, and inhibition

**DOI:** 10.1111/apha.14016

**Published:** 2023-06-27

**Authors:** Sotiria Tavoulari, Maximilian Sichrovsky, Edmund R. S. Kunji

**Affiliations:** ^1^ Medical Research Council Mitochondrial Biology Unit University of Cambridge Cambridge UK

**Keywords:** metabolism, mitochondria, pyruvate transport, small molecule inhibitors, transport mechanism

## Abstract

The mitochondrial pyruvate carrier (MPC) resides in the mitochondrial inner membrane, where it links cytosolic and mitochondrial metabolism by transporting pyruvate produced in glycolysis into the mitochondrial matrix. Due to its central metabolic role, it has been proposed as a potential drug target for diabetes, non‐alcoholic fatty liver disease, neurodegeneration, and cancers relying on mitochondrial metabolism. Little is known about the structure and mechanism of MPC, as the proteins involved were only identified a decade ago and technical difficulties concerning their purification and stability have hindered progress in functional and structural analyses. The functional unit of MPC is a hetero‐dimer comprising two small homologous membrane proteins, MPC1/MPC2 in humans, with the alternative complex MPC1L/MPC2 forming in the testis, but MPC proteins are found throughout the tree of life. The predicted topology of each protomer consists of an amphipathic helix followed by three transmembrane helices. An increasing number of inhibitors are being identified, expanding MPC pharmacology and providing insights into the inhibitory mechanism. Here, we provide critical insights on the composition, structure, and function of the complex and we summarize the different classes of small molecule inhibitors and their potential in therapeutics.

## 1 INTRODUCTION

Mitochondria generate most of the chemical energy needed for cellular function and play a fundamental role in many cellular processes and biosynthetic pathways. To achieve this, metabolites and ions are exchanged between the cytoplasm and mitochondrial matrix, crossing two mitochondrial membranes. Ions and small molecules can permeate the outer mitochondrial membrane via pore‐forming voltage‐dependent ion channels, but the inner mitochondrial membrane is impermeable, requiring specific mitochondrial transporters and channels for their translocation. The majority of transporters belong to the SLC25 mitochondrial carrier family, comprising of 52 members that transport amino acids, sugars, nucleotides, products of fats, vitamins, and inorganic ions.^1^ With the exception of the aspartate/glutamate carrier,^2^ the vast majority of the SLC25 family members are monomeric^1^, ^3^ and have three homologous domains, each composed of two transmembrane α‐helices linked with a loop and short α‐helix on the matrix side.^4^ The mitochondrial ADP/ATP carrier and the uncoupling protein, which both belong to the SLC25 family, are the only functional carriers that have been structurally characterized.^5^, ^6^, ^7^, ^8^ However, there is limited knowledge about the structure and mechanism of other transporter families in mitochondria.

The mitochondrial pyruvate carrier (MPC) mediates the entry of pyruvate into the mitochondrial matrix, achieving a crucial role in linking glycolysis to oxidative phosphorylation and therefore, cytosolic to mitochondrial metabolism. When pyruvate produced in glycolysis is transported into the mitochondrial matrix by the MPC it can be oxidized by converting to acetyl‐coenzyme A (acetyl‐CoA), generating an NADH molecule from NAD^+^. Acetyl‐CoA then enters the tricarboxylic acid cycle (TCA cycle), which leads to production of one GTP, which can be converted to ATP, and three NADH molecules. Electrons from NADH are transferred to the respiratory chain by complex 1. The redox reactions in complexes 1, 3, and 4 are coupled to the extrusion of protons from the mitochondrial matrix to the intermembrane space, generating a proton motive force used by ATP synthase to drive the synthesis of ATP from ADP and inorganic phosphate. Overall, pyruvate oxidation via the TCA cycle and the respiratory chain reactions yields up to 36 molecules of ATP.

The existence of a mitochondrial protein responsible for this function was demonstrated back in the 1970s, as pyruvate transport displayed saturation kinetics, was sensitive to sulfhydryl reagents^9^ and small molecules could block mitochondrial pyruvate import.^10^ Despite these early studies, the identification of the MPC proteins was only achieved in 2012.^11^, ^12^ It was shown that there are several isoforms per species that are involved in the transport of pyruvate in mitochondria. These proteins were originally shown to form large hetero‐complexes of ~150 KDa^12^ on blue native electrophoresis, but this approach leads to artifacts for small membrane proteins.^13^ Recently, it was demonstrated that the functional MPC is hetero‐dimeric.^14^, ^15^ Despite its name, the MPC is unrelated to SLC25 members and was assigned to a new protein family, named SLC54.^16^

Given its central role in metabolism, MPC has been proposed as a pharmacological target for different pathologies, including diabetes, non‐alcoholic steatohepatitis (NASH),^17^, ^18^, ^19^, ^20^, ^21^ neurodegenerative disorders,^22^, ^23^, ^24^ and specific cancers.^25^, ^26^, ^27^ An increasing number of studies have been addressing the physiological consequences of MPC inhibition. The composition, oligomeric state and functional reconstitution of MPC have also been described,^14^, ^15^ providing the platform to elucidate the mechanistic details. However, the molecular mechanisms of transport and inhibition are poorly defined. In this review, we compile past and present findings on the structure, function and inhibition of the MPC as well as the therapeutic potential of MPC inhibition.

## 2 THE INVOLVEMENT OF MPC IN METABOLISM

**FIGURE 1:**
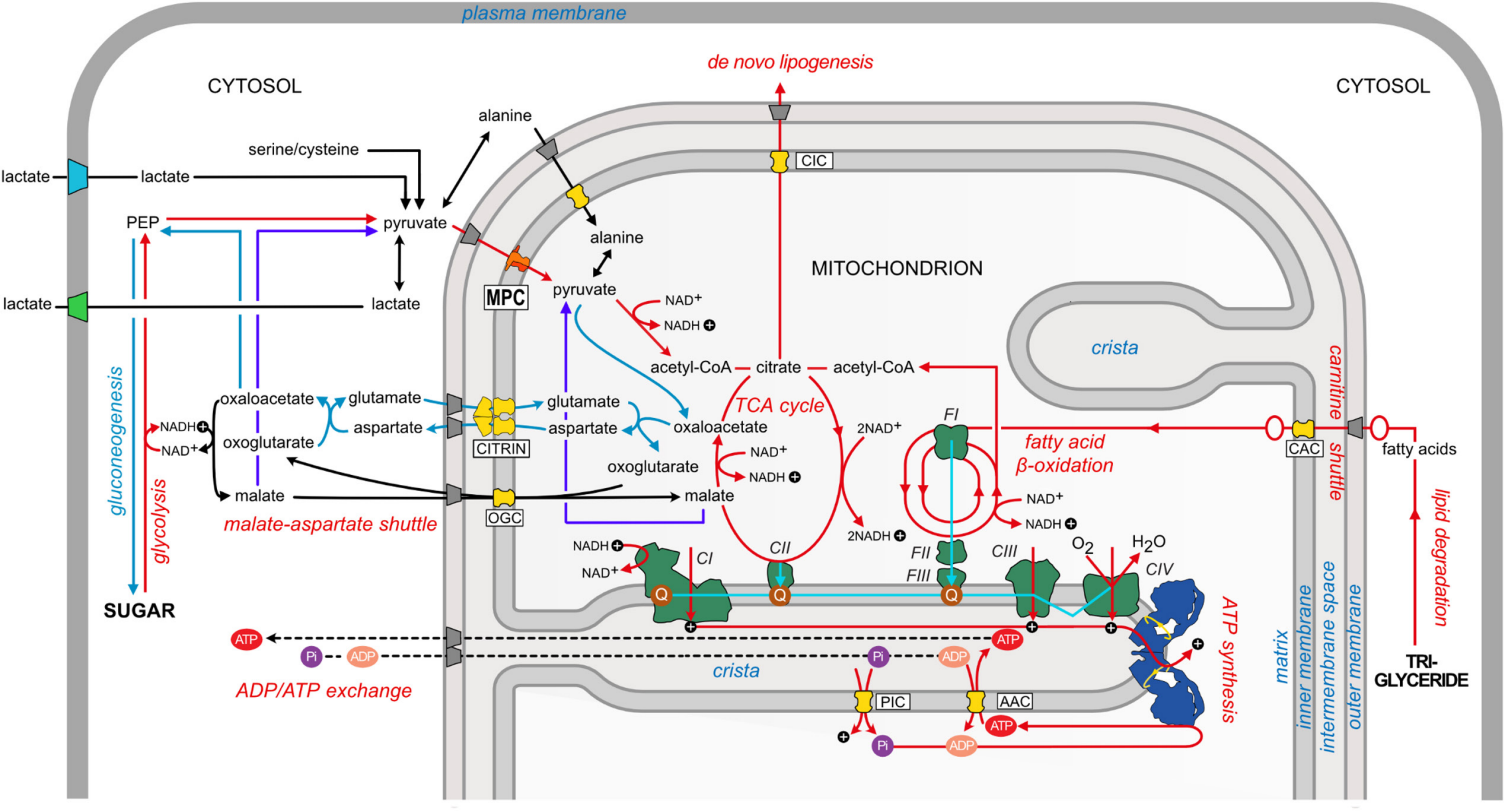
Biochemical pathways involved in glycolysis (red), tricarboxylic acid cycle (TCA) (red), respiratory chain (cyan), gluconeogenesis (blue), malate–aspartate shuttle (black), de novo lipogenesis and beta oxidation (red) are shown schematically. Conversion of malate to pyruvate is shown in purple. The MPC dimer is shown in red/orange and other mitochondrial carriers in yellow (aspartate/glutamate carrier (citrin), ADP/ATP carrier (AAC), carnitine/acylcarnitine (CAC), phosphate carrier (PIC), and oxoglutarate carrier (OGC)). The respiratory chain complexes 1–4 (CI–CIV) and acyl‐CoA dehydrogenases (FI), electron transfer flavoprotein (FII), and ETF‐ubiquinone oxidoreductase (FIII) are shown in green and the dimer of ATP synthase in blue. The voltage‐gated anion channel (VDAC) in the outer membrane is shown in gray. Key metabolites, such as phosphate (Pi, purple), ADP (orange), ATP (red) and ubiquinone (Q, brown) are shown, and protons are black circles with a plus sign.

The MPC is one of the major gatekeepers of pyruvate metabolism. By allowing pyruvate entry into mitochondria, it regulates cellular homeostasis, as this key metabolic step links aerobic and anaerobic metabolism and is involved in several important pathways, as described below. The major source of pyruvate in the cell is glycolysis, where in the last step phosphoenolpyruvate is converted to pyruvate via pyruvate kinase. Other sources of pyruvate are the oxidation of lactate via lactate dehydrogenase (LDH), malate via malic enzymes, alanine via alanine transaminase (ALT), or other gluconeogenic amino acids (Figure 1). Here, we will briefly summarize the different fates of pyruvate under different metabolic conditions, as they have been reviewed extensively elsewhere.^28^, ^29^

Once formed, pyruvate can be fully oxidized in mitochondria, after it is transported into the mitochondrial matrix by the MPC. First, the pyruvate dehydrogenase complex (PDC) generates acetyl‐CoA, which then combines with oxaloacetate (OAA) to form citrate, the first substrate of the TCA cycle, ultimately fueling oxidative phosphorylation and ATP production. ATP levels, the metabolic content and the redox state of the cell regulate PDC and the TCA cycle by glucose‐derived pyruvate. Citrate produced from pyruvate can be exported from the mitochondrion, where it is converted to acetyl‐CoA, the building block for de novo lipogenesis, thereby linking MPC to fatty acid synthesis.

Pyruvate that is not used for the synthesis of acetyl‐CoA can be converted directly to OAA by pyruvate carboxylase (PC). This fate is also controlled by MPC as PC resides in the mitochondrial matrix and the conversion requires pyruvate import into the mitochondrion. Importantly, OAA is a precursor for several non‐TCA cycle related pathways. In certain tissues, such as the liver, more pyruvate is converted to OAA than to acetyl‐CoA.^30^ In this way, OAA can enter gluconeogenesis in the liver in order to maintain plasma glucose levels during fasting and it contributes to the synthesis of glucagon and amino acids (Figure 1). Overall, pyruvate import in mitochondria is critical for replenishing TCA cycle intermediates, for gluconeogenesis but also for other pathways, including the malate/aspartate shuttle, the urea cycle via OAA and lipid synthesis via citrate. Pyruvate is also a direct precursor of alanine during amino acid biosynthesis. Alanine is produced via a transamination reaction between pyruvate and glutamate by the alanine aminotransferase/glutamic‐pyruvate transaminase (ALT/GPT).

Pyruvate has a different fate under anaerobic conditions, as in the exercising muscle tissue, when it can be reversibly reduced to lactate via LDH in the cytosol, converting NADH to NAD^+^ to stimulate glycolysis. Lactate produced in the muscle under these conditions can then be excreted, enter the circulation and be converted back to pyruvate in the liver. With intense exercise, high levels of lactate can accumulate in the muscle causing lactic acidosis. In intestinal flora and in fermenting yeast, pyruvate can also be converted to acetaldehyde and then ethanol.

### 2.1 Metabolic consequences of MPC deletion or pharmacological inhibition

In the past decade, numerous studies have addressed the role of MPC in pathophysiology via pharmacological inhibition or genetic ablation and these have been reviewed extensively elsewhere.^31^, ^32^ MPC is critical for embryogenesis and mice do not survive constitutive deletion of either protomer, MPC1 or MPC2.^33^, ^34^ However, whole body Mpc1+/− mice^35^, ^36^ as well as tissue specific knock‐outs (KO) and pharmacological MPC inhibition have been performed in several tissue types, including liver^37^, ^38^ skeletal muscle,^39^ heart,^40^, ^41^, ^42^ and retina.^43^ The consequences of altered MPC activity vary dramatically between tissues and cell types, and it is currently unclear whether therapeutic interventions targeting MPC will prove beneficial. What is evident is that most commonly, metabolic plasticity follows MPC inhibition or deletion, allowing cells to compensate for carrier loss in a tissue‐specific manner.

In the liver, where pyruvate conversion to OAA is substantial and important for gluconeogenesis, MPC liver‐specific loss results in reduced gluconeogenesis, but triggers an adaptive mechanism where gluconeogenesis is sustained by the amino acids alanine and glutamine.^37^, ^38^ Since MPC inhibition or deletion reduces rates of gluconeogenesis, it protects mouse models of insulin resistance and type II diabetes from hyperglycemia.^37^, ^38^, ^44^ Concomitantly, in humans, new generation insulin sensitizers MSDC‐0160 and MSDC‐0602K targeting MPC have been in clinical trials for type II diabetes.^45^

The pharmacological inhibition of MPC in the liver is also relevant to new therapeutic strategies for NASH.^18^, ^21^ The liver is essential for lipid metabolism and it has been shown that hepatocyte‐specific MPC2 deletion and pharmacological inhibition of MPC with MSDC‐0602K in a mouse model of NASH reduced liver injury, fibrosis and hepatic stellate cells activation.^17^ In MPC1 KO mice, protection was shown against NASH development after a long‐term high fat diet.^46^ Additionally, pharmacological inhibition of MPC in primary hepatocytes decreased liver inflammation.^46^

A big challenge following MPC inhibition is energy output, as the TCA cycle is not supported by pyruvate oxidation under this condition. Different studies have shown that compensatory pathways do exist and retain TCA cycle activity and energy production to some extend by increasing glutaminolysis and oxidation of fatty acids in the liver,^38^ brown adipose tissue,^36^, ^47^, ^48^ skeletal muscle,^39^, ^49^ and the heart.^41^, ^42^ However, these mechanisms are not always sufficient. Although basal muscle function is retained with MPC loss, when exercise is energetically more demanding the compensatory mechanisms are not sufficient and mice present exercise intolerance. More importantly, a big challenge is the effect on the heart, which is one of the most energetically demanding tissues. In the heart, fatty acid oxidation accounts for the majority of energy production, with glucose oxidation and oxidative phosphorylation contributing less. Although increased fatty acid oxidation compensates for loss in mitochondrial pyruvate oxidation in the healthy heart, MPC loss in mouse heart has been shown to drive heart failure^41^, ^42^, ^50^ which can be rescued by ketogenic diet.^50^

## 3 COMPOSITION AND OLIGOMERIC STATE OF THE MPC FUNCTIONAL UNIT

Seminal work from the laboratories of Jean‐Claude Martinou and Jared Rutter led to the identification of proteins responsible for pyruvate transport in the mitochondria of yeast, flies and humans.^11^, ^12^ Surprisingly, it was found that MPC is not a single protein, but consists of two small homologous membrane proteins that form hetero‐complexes.^11^, ^12^ Yeast *S. cerevisiae* expresses three proteins, Mpc1, Mpc2, and Mpc3, with molecular weights of ~15, ~15.5, and ~17 KDa, respectively. The carbon source availability defines which complex is predominantly expressed, with Mpc1/Mpc2 complex forming in fermentative conditions and Mpc1/Mpc3 in respiratory conditions.^51^ In humans there are also three MPC proteins, MPC1 (~12 KDa), MPC2 (~14 KDa), and MPC1L (~15 KDa), the latter being homologous to MPC1. Two alternative complexes are formed, one between MPC1 and MPC2, ubiquitously expressed in all tissues, and a separate one, expressed in the testis, between MPC1L and MPC2.^52^ As with yeast, there are more than two MPC protomers expressed in plants, however the biological functions and molecular mechanisms surrounding MPC in plants are not well understood. In *Arabidopsis thaliana*, there are at least four MPC isoforms, MPC1, MPC2, MPC3, and MPC4. MPC1 has been shown to interact with MPC3 (previously named NRGA1) via a co‐immunoprecipitation assay.^53^ Deletion of MPC1 causes concomitant loss of MPC3 and MPC4, in addition to a loss of pyruvate transport in *Arabidopsis thaliana* mitochondria^54^ but it is unknown if MPC3 and MPC4 are able to form interactions in the absence of MPC1.

The composition, size, and oligomeric state of the MPC complexes have been investigated in different ways. Using blue native gel electrophoresis Bricker and co‐workers originally proposed a complex of 150 KDa between *S. cerevisiae* Mpc1 and Mpc2.^12^ However, the electrophoretic mobility of membrane proteins on blue native gels is affected by detergents and lipids that remain associated with the protein, when it is extracted from the membrane, increasing the size of the species. Since Coomassie stain binds to the associated detergent–lipid micelle as well as to the protein, the migration on the gel does not reflect the molecular mass of the protein, rendering this technique problematic for estimating the molecular mass of small membrane proteins.^13^ A different biochemical approach used 7 Å‐ or 15 Å‐long chemical cross‐linkers and the cross‐linked human MPC appeared in multiple bands in gels, corresponding to monomers, dimers and higher molecular weight oligomers.^51^, ^55^ However, some of the different oligomeric states could be the result of non‐native crosslinking events and thus might not be relevant to functional MPC.

**FIGURE 2:**
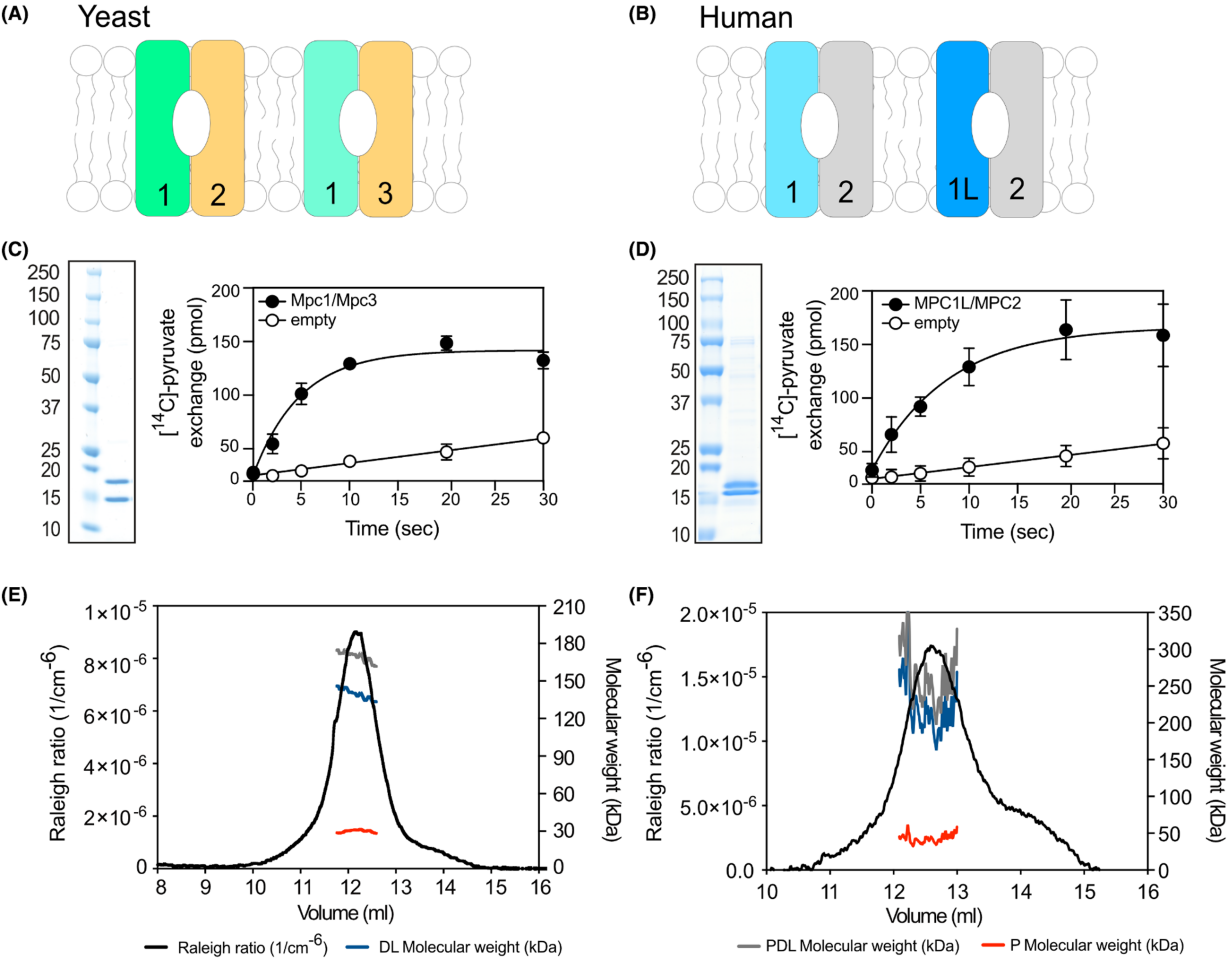
Schematic representation of the functional yeast (A) and human (B) mitochondrial pyruvate carrier (MPC) dimers. Pyruvate transport activity measured in yeast Mpc1/Mpc3 (C) or human MPC1L/MPC2 (D) proteoliposomes in comparison with empty liposomes at a ΔpH of 1.6. Oligomeric state analysis for the purified yeast Mpc1/Mpc3 (E) and human MPC1L/MPC2 (F) by SEC‐MALLS. In (E) and (F), the masses of the protein–detergent–lipid complex (PDL, gray), the detergent–lipid micelle (DL, blue), and the protein (P, red) are indicated.

We followed a biophysical approach to determine the molecular mass, oligomeric state, and subunit stoichiometry of MPC by using the purified yeast Mpc1/Mpc3 and human MPC1L/MPC2 hetero‐complexes, analyzed via size‐exclusion chromatography linked to multi‐angle laser light scattering (SEC‐MALLS).^14^, ^15^ This technique can determine the mass of the detergent–lipid complex and the protein component^56^, ^57^ and therefore, is considered the golden standard for sizing of membrane proteins. The results showed that the human and yeast complexes, with the two protomers purified in a 1:1 stoichiometry, form hetero‐dimers that mediate pyruvate transport (Figure 2) and can bind known MPC inhibitors with the expected affinities.^15^ Hence, we concluded that the functional unit of MPC is a hetero‐dimer.

In separate studies, oligomers of the human MPC2 have been proposed to be active for pyruvate transport as an autonomous entity.^55^ A number of findings argue against this proposal, showing that MPC1 is also necessary for the formation of the functional complex, for substrate translocation and inhibitor binding. First, mitochondria lacking MPC1 were unable to import pyruvate.^11^, ^12^ Additionally, mutations Leu79His, Arg97Trp, Ala32Gly and Lys72Glu in MPC1 have been described as pathogenic and cause pyruvate deficiency.^12^, ^58^, ^59^ Recently, Hegazy and co‐workers also described that MPC1 mutation Phe66Ala impairs substrate and inhibitor binding.^60^ Most importantly, human MPC2 homomers did not bind the classic MPC inhibitors, such as (2E)‐2‐Cyano‐3‐(1‐phenyl‐1H‐indol‐3‐yl)acrylic acid (UK5099), with the expected affinities, implying that they do not form a functional binding pocket.^55^, ^61^

## 4 STRUCTURAL MODELS

No experimentally determined structure is currently available for the MPC, which is most likely related to difficulties associated with purification and stabilization of these small and dynamic hetero‐dimeric complexes. However, proposals have been made for the topology of the protomers and structural models are being developed based on computational approaches.

An early study on yeast MPC addressed the topology of each protomer.^51^ Single cysteine variants of yeast Mpc1 and Mpc3 were engineered and expressed in a yeast null background and mitochondria were labeled with a 0.5 KDa thiol‐reactive dye, which preferentially labels cysteines in the intermembrane space but not in the matrix. The results suggested that Mpc1 consists of only two transmembrane helices, whereas Mpc3 has three, and that both termini of yeast Mpc1 face the mitochondrial matrix, while Mpc3 should have an N‐terminus facing the matrix and the C‐terminus the intermembrane space. However, this topology contradicts hydropathy profiles, secondary structure predictions and computational models, which are described below. Moreover, it is unlikely that these two homologous proteins with high levels of identical and similar residues have different topologies. The observation that Mpc3 can form both a functional hetero‐dimer with Mpc1 and a non‐functional homo‐dimer also indicates that these proteins must have similar structures.^14^ The discrepancies between the experimental and sequence‐based topologies could be caused by technical issues with the labelling technique, as the low molecular weight of the dye resulted in minimal migration differences between labeled and unlabeled variants.^51^

**FIGURE 3:**
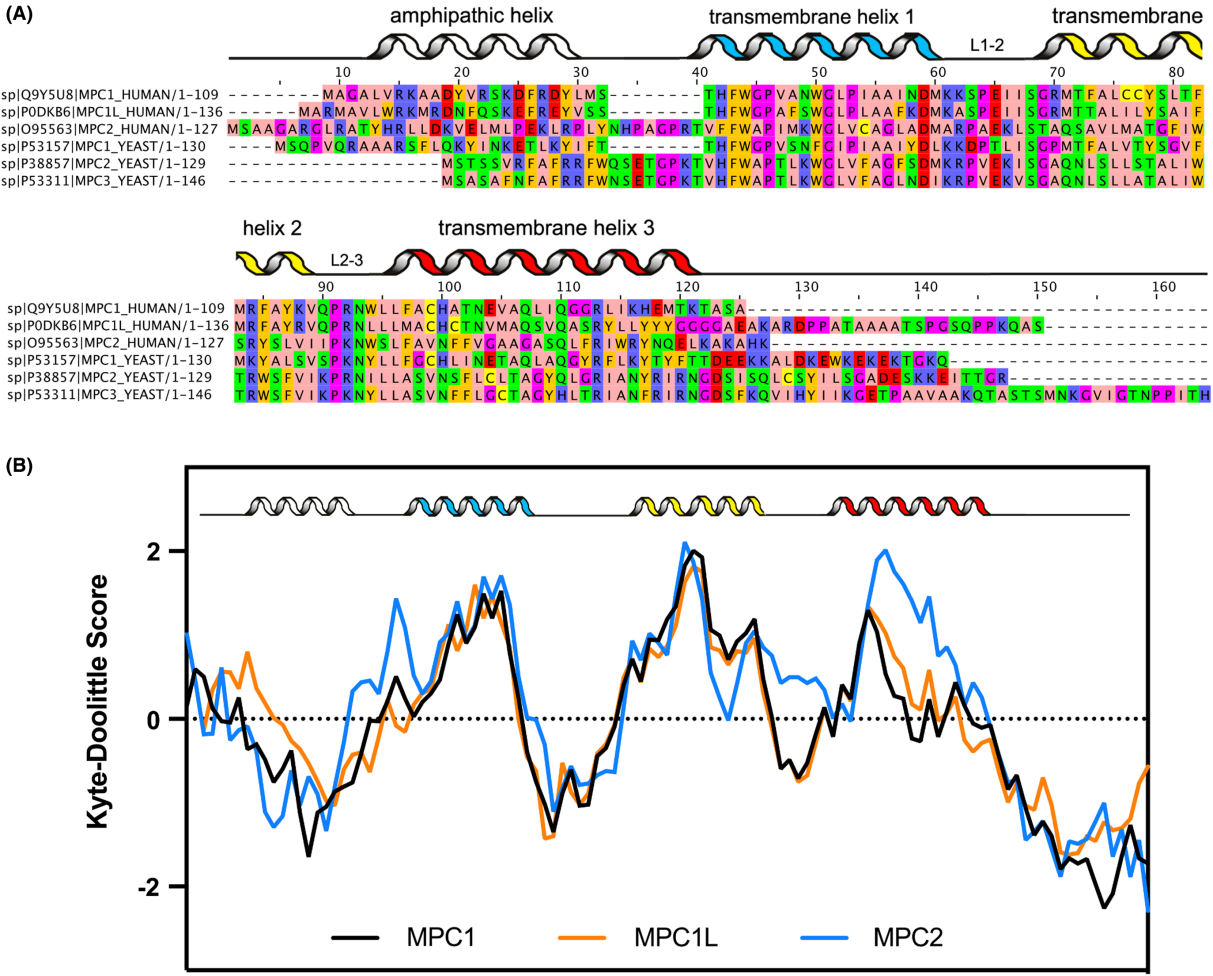
(A) Sequence alignment of human and yeast MPC proteins, generated by Jalview.^103^ The residues are labeled according to the Zappo coloring scheme, where aliphatic, polar, aromatic, positively charged, negatively charged, Pro/Gly, and Cys, are colored pink, green, orange, blue, red, magenta, and yellow, respectively. (B) Average hydropathy score for each position across MPC proteins from different species; MPC1 (*n* = 96), MPC2 (*n* = 132), and MPC1L (*n* = 60). Sequences were aligned using Jalview and each residue was given a hydropathy score following the Kyte–Doolittle matrix.^104^ Moving averages across 16 residues were used to calculate the final score for each position, after which all three plots were aligned according to position of the predicted transmembrane helices. Portions after the final transmembrane helix were truncated in each alignment. The secondary structure predictions given are based on AlphaFold structural models, as well as the hydropathy plots.

What has been established as a consensus in the literature, but still requires experimental validation, is that MPC is a structural homologue of the bacterial semiSWEET transporters,^62^ which are homo‐dimers. This hypothesis relies on algorithms predicting one N‐terminal amphipathic helix and three transmembrane helices for each MPC protomer, which are also observed in the semiSWEETs.^63^ Figure 3 shows the hydropathy profiles of the yeast and human MPC protomers supporting this prediction, as they show three clear hydrophobic regions in the protein, representing each transmembrane helix. The third helix (TM3) shows the weakest overall hydrophobicity of the three, which is possibly a result of the C‐terminal portion of the helix extending beyond the membrane. Atomic structures of the SemiSWEET homo‐dimer have been solved by X‐ray crystallography in different conformational states,^63^ demonstrating an alternating access mechanism of sugar transport.^64^ More recently, the structure of the MPC protomers was also predicted by Deepmind AlphaFold, showing similar structural features.^65^, ^66^

Three different groups have presented structural models of MPC homo‐ or hetero‐dimers based on structural alignment with the semiSWEETs^61^, ^67^ or de novo models using either trRosetta^67^ or Alphafold.^60^ Using semiSWEET transporters as a template, Lee and co‐workers proposed a homology model for the hetero‐dimer with inverted topology for the two protomers and identified highly conserved residues in human MPC1 and MPC2 in the proposed dimeric interface. Xu et al. presented de novo models of the MPC1 and MPC2 using Rosetta and introducing an artificial linker between protomers. The most energy favorable model was produced when the N‐terminal amphipathic helices from each protomer were positioned parallel to the membrane and in the matrix side, and the C‐terminals of the protomers were facing the intermembrane space. The resulting model adapted a similar overall topology as the semiSWEET homo‐dimers, although the positions of the transmembrane segments were different. However, neither model was experimentally validated. More recently, Hegazy and co‐workers have used a model based on the AlphaFold structural predictions of the protomers. The rational for the dimer assembly was not described, but a small number of residues with side chains facing into the dimer interface were mutated to alanine and inhibitor binding studies were performed.^60^ The results suggested that Phe66 in MPC1 and Asn100 and Lys49 in MPC2 are important for inhibitor binding. This is consistent with our proposal that both protomers are involved in inhibitor binding.^15^

**FIGURE 4:**
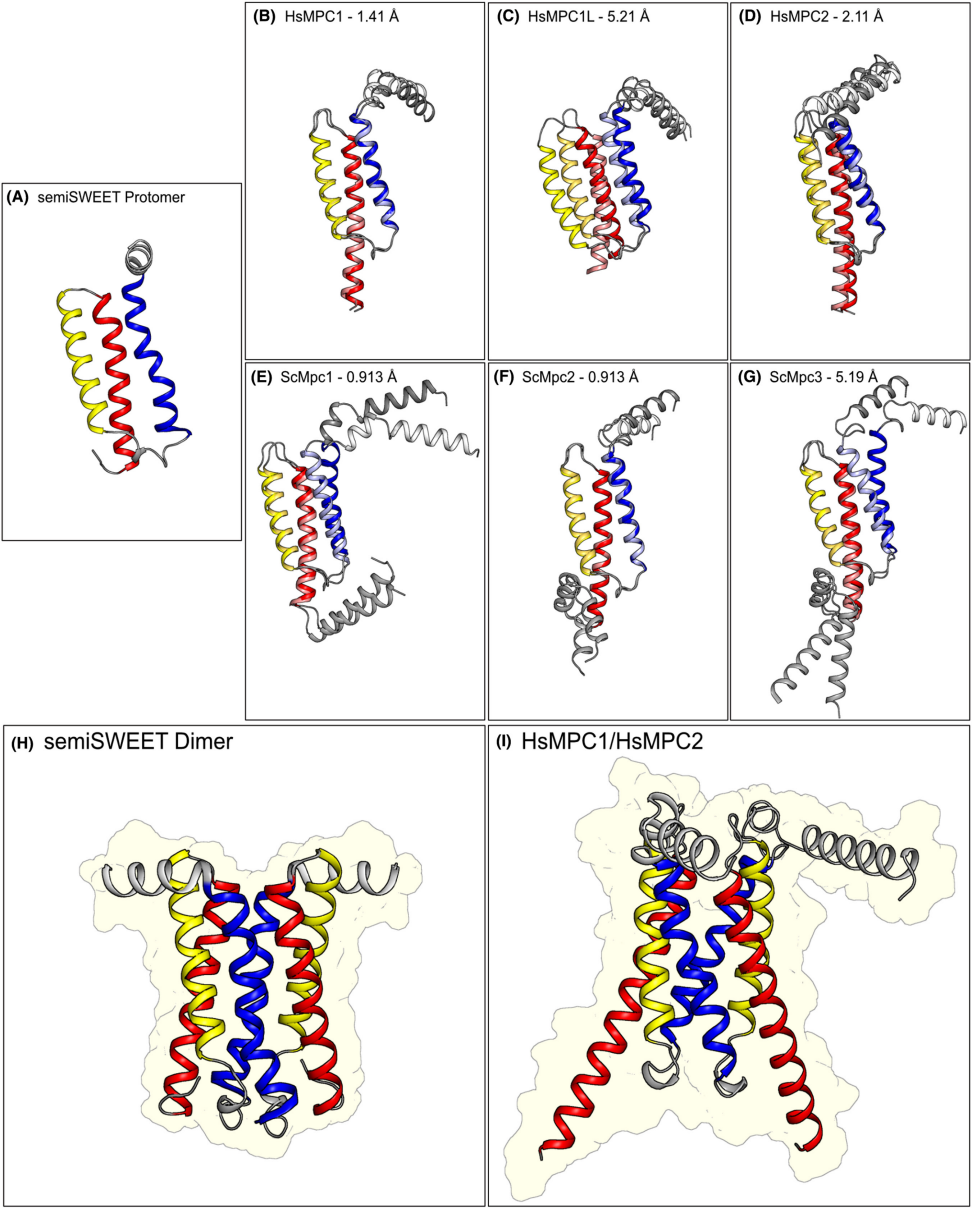
Comparison of MPC with the SemiSWEET transporter. (A) *Vibrio sp* SemiSweet transporter protomer structure (PDB: 4QND). (B–G) Structural predictions of each human and yeast MPC protomer. AlphaFold predictions^65^, ^66^ are shown in bold colors, while predictions from trRosetta^105^, ^106^ are shown in lighter shades of the same color‐scheme. In (H, I), the homo‐dimer of the SemiSWEET is shown, as well as a predicted structure of the hMPC1/MPC2 hetero‐dimer. In all panels, transmembrane helices TM1, TM2, and TM3 are shown in blue, yellow, and red, respectively, with the N‐terminal amphipathic helix shown in white. The C‐terminal extensions in yeast MPC proteins, predicted to be helical, are shown in gray. Root mean square deviation values, comparing the predictions by AlphaFold and trRosetta, are indicated.

We have performed comparative analysis of the structural models from AlphaFold and trRosetta for the yeast and human MPC protomers (Figure 4). The comparison shows that the agreement on the three‐transmembrane‐helical topology of each protomer is very high. Discrepancies in the exact position of each transmembrane helix and the amphipathic helix are responsible for higher root mean square deviation values seen in hMPC1L and ScMPC3. Since the membrane context is not used in the structure prediction software, it is expected that the position of the amphipathic helix could be inconsistent. In addition, the C‐terminal region of hMPC1L is predicted to be a long and disordered loop, and it was therefore truncated for this analysis. C‐terminal extensions are also present in the yeast protomers, which are predicted by both software as alpha‐helical. In ScMPC2 and ScMPC3, these additional helices are separated from the third transmembrane helix (TM3) by a short helical turn, also seen separating the amphipathic and transmembrane helix 1 (TM1) of hMPC1, hMPC1L, hMPC2, and ScMPC2. Subsequently, we have used the AlphaFold models of the two human protomers, MPC1 and MPC2, to assemble the hetero‐dimer guided by the outward‐facing structure of the semi‐SWEETs (Figure 4).

Overall, there is significant agreement between algorithms supporting structural homology of the protomers with the semiSWEETs, but experimental validation is missing and several questions are outstanding related to the assembly of the hetero‐dimer, including the topology of the two protomers, their relative position within the dimer, as well as the position of the amphipathic helices. Although structural analysis will be necessary to answer these questions unequivocally, biochemical and biophysical techniques are now available to facilitate validation of work models.

## 5 MECHANISM OF PYRUVATE TRANSPORT BY THE MPC

The first hypothesis was that pyruvate can enter mitochondria via diffusion in its undissociated form.^68^, ^69^ However, Papa and colleagues showed that pyruvate translocation and exchange displayed saturation kinetics and was sensitive to sulfhydryl reagents, supporting the existence of a pyruvate transporter.^9^ Critical evidence was also provided by the lab of Andrew Halestrap, who identified a small molecule that could inhibit pyruvate oxidation by blocking mitochondrial pyruvate transport in whole but not in ruptured mitochondria.^10^ A decade after the molecular identification of MPC proteins, little is known about the mechanism of transport.

An important aspect of pyruvate flux in mitochondria is that it is driven by the ΔpH. This was first proposed in the 70s in isolated rat mitochondria^9^, ^70^, ^71^ and we have recently confirmed unequivocally in proteoliposomes of purified yeast and human MPC hetero‐dimers.^14^, ^15^ It is also now understood that two MPC protomers within the functional dimer are necessary for substrate transport^11^, ^12^, ^14^, ^52^ but several outstanding questions remain, including the molecular nature of the ΔpH‐dependency and the location of the substrate binding site.

Interestingly, studies in isolated mitochondria suggest that MPC could have a rather broad substrate specificity and can exchange pyruvate for a range of substrates, including oxamate, 2‐oxobutyrate, phenylpyruvate, 2‐oxo‐4‐methyl‐pentanoate, chloroacetate, dichloroacetate, difluoroacetate, 2‐chloropropionate, 3‐chloropropionate, and 2,2‐dichloropropionate.^71^, ^72^ The maximum rate of transport is affected not just by the ΔpH, but also by the presence of exchangeable substrates.^72^ This observation opens the possibility that MPC works both as an exchanger and a uniporter, depending on the metabolic content and the substrate availability. This could be of great physiological importance, especially if MPC could be exchanging pyruvate for ketone bodies under ketogenic conditions. However, a ketogenic diet rescued the phenotype in mice with conditional deletion of MPC1 in glutamatergic neurons, suggesting that ketone body import in mitochondria could be MPC‐independent.^73^ The ability of MPC to exchange pyruvate for ketone bodies has not been verified by transport assays with the reconstituted hetero‐dimer.

## 6 FUNCTIONAL ASSAYS AVAILABLE FOR THE MPC

The first studies on the function of MPC had been conducted in the 1970s using isolated mitochondria from mammalian species. Several findings have now been confirmed and some have been disputed, but the progress made then remains remarkable, considering that the proteins had not been identified and the available experimental systems at the time were limited. The molecular identification and the potential of MPC as a drug target sparked additional interest to establish new functional assays, and, here, we summarize and compare the most important ones that measure transport or binding on MPC.

### 6.1 Substrate transport and transport inhibition in isolated mitochondria

This is the typical method used in the original work for MPC functional characterization. Isolated mitochondria from mammalian species were incubated in buffer containing radiolabeled pyruvate, which gets transported into the matrix by MPC. The reaction was stopped either by pelleting mitochondria via centrifugation^70^, ^72^ or by filtration through a membrane to separate mitochondria from the uptake buffer.^74^ After the high affinity inhibitors a‐cyano‐4‐hydroxycinnamate (CHC) and UK5099 were identified, they were used for rapid termination of transport.^10^, ^71^ More recently, an advanced version of this technique has allowed these measurements to be carried out in a 96‐well format.^75^ The advantage of a technique using isolated mitochondria is that MPC is assayed in the native membrane environment, but it is more challenging to control the transport conditions and to interpret the data. First, the isolation of intact mitochondria is not trivial and second, the ongoing pyruvate oxidation can hinder the detection of the imported pyruvate.^71^ As a result, these experiments are usually conducted at 4–6°C,^71^, ^74^ rather than physiologically relevant temperatures, which is expected to reduce the transport rates. Another major drawback, however, is that the dense mitochondrial inner membrane contains a great number of other carriers, making the specificity of tested effectors on MPC hard to establish.

### 6.2 Pyruvate transport and inhibition in the bacterium *Lactococcus lactis*

Pyruvate transport was previously measured in the bacterium *Lactococcus lactis* where both mouse MPC protomers, MPC1 and MPC2 were expressed.^11^ A fourfold increase in pyruvate uptake was detected when mMPC1 and mMPC2 were co‐expressed, whereas no pyruvate uptake was observed in bacteria expressing either protein alone. Transport by the mMPC1/MPC2 complex was inhibited by UK5099. However, the time course of pyruvate transport in *L. lactis*, up to 1 h in room temperature, is unexpected and incompatible with the fast time course reported for MPC in isolated mitochondria^71^, ^75^ as well as in MPC proteoliposomes.^15^ Additionally, the rates of transport were about one thousand times lower compared to transport rates observed in proteoliposomes.^15^ These results imply that the reported pyruvate accumulation in *L. lactis* is sub‐optimal for validation of its transport properties.

### 6.3 Substrate transport and transport inhibition in proteoliposomes prepared with purified protein

A robust way to study transport is via reconstitution of purified protein into liposomes, which became possible only after the molecular identification of MPC. We have established this approach and have assessed transport of pyruvate for human hetero‐dimers and yeast homo‐ and hetero‐dimers in phosphatidylcholine (PC)/cardiolipin (TOCL) liposomes.^14^, ^15^ Human MPC2 homomers have been studied in asolectin liposomes.^55^ As soon as the proteoliposomes are formed, the method relies on radiolabeled substrate transport followed by rapid filtration of the liposomes to separate them from the external substrate and subsequent quantification by scintillation counting, which makes the method highly sensitive. The advantages of this approach are that chemical gradients are easier to impose and control, any effectors can be tested directly on the MPC protein and robust quantitative data can be collected for mechanistic studies or inhibitor screening. Since the protein is in an artificial membrane bilayer, one can argue that the activity is not comparable to what is measured in native membranes. However, the kinetic parameters we calculated for the human MPC hetero‐dimer in PC/TOCL^15^ match very well with those observed in studies with isolated mitochondria.^71^ The requirement for purified protein, which is technically challenging because of the labile hetero‐dimer, is a disadvantage.

Pyruvate transport assays, both in isolated mitochondria and proteoliposomes, face common challenges, such as the instability and oligomerization of pyruvate and the ability of pyruvate to diffuse through membranes at low pH values.

### 6.4 Assessment of MPC transport via pyruvate biosensors

MPC activity was recently assessed via measurements of pyruvate concentrations within the cell using pyruvate biosensors.^76^, ^77^ PyronicSF is a pyruvate biosensor proposed to enable real‐time subcellular quantitation of pyruvate concentration and flux.^76^ In vitro, the Kd of the biosensor for pyruvate is estimated at 480 μM. PyronicSF can be targeted to the mitochondrial matrix using the mitochondrial target sequence of cytochrome oxidase and is insensitive to lactate and other structurally related organic acids known as putative MPC substrates, but it is moderately affected by pH. Although the uptake of pyruvate by mitochondria, as measured via PyronicSF, was sensitive to the MPC inhibitor UK5099, the inhibition was not complete even at 10 μM, although the Ki of UK5099 has been determined to 50 nM. This result implies that the biosensor could also be measuring alternative routes of pyruvate uptake. The advantage of the technique is that it can be used in situ and only requires standard wide‐field and confocal microscopes.

### 6.5 Bioluminescence resonance energy transfer (BRET) ligand‐binding assay

In this assay, developed in the lab of Jean‐Claude Martinou, the C‐termini of the two MPC protomers, MPC1 and MPC2, are fused to either RLuc8 (donor group) or Venus (acceptor group), or other donor/acceptor pairs, and expressed in mammalian cells.^78^ The technique relies on the idea that conformational changes will occur during ligand binding to alter the proximity between the termini and will therefore, change the energy transfer between MPC1 and MPC2. The advantage of the technique is that it detects MPC activity in situ and in real time, but the termini are artificially elongated, which may not necessarily reflect the natural movements in response to binding in the binding pocket.

### 6.6 Thermostability shift assays to evaluate binding

We have employed two alternative thermostability shift assays to monitor ligand binding to purified MPC.^15^ The general idea is that when a folded protein is subjected to a temperature ramp it starts unfolding and if this is coupled to detectable changes, an apparent melting temperature for the population of proteins can be calculated.^79^, ^80^ Changes in the melting temperature can be observed upon ligand binding, because additional interactions with the protein occur.^79^, ^81^ One type of thermostability shift assay we used for MPC relies on the reaction of endogenous cysteines with 7‐diethylamino‐3‐(4‐maleimidophenyl)‐4‐methylcoumarin (CPM).^14^, ^15^ As MPC unfolds in the temperature ramp, cysteines originally buried become solvent‐exposed and react with CPM to form fluorescent adducts and the fluorescence increase is monitored throughout the temperature ramp. The second assay is the dye‐free nano differential scanning fluorimetry (nano‐DSF), which monitors fluorescence changes due to changes in the environments of tryptophan and tyrosine residues during unfolding.^14^ The major advantage of these assays is the high‐throughput nature, the direct assessment of binding on purified MPC and the low amounts of protein that are required. However, the requirement of protein purification makes it costly and binding parameters cannot be determined directly. Occasionally, there could also be false negatives. Lonidamine, which inhibits pyruvate transport with low micromolar potency, does not yield a thermal shift in the two thermostability assays.^15^

## 7 THE VARIOUS MPC INHIBITORS AND THEIR PROPOSED THERAPEUTIC USES

The first MPC inhibitors were identified by the Halestrap group long before the molecular identification of the protein complex. Using isolated rat liver mitochondria, CHC was first shown to reduce pyruvate oxidation and pyruvate transport.^10^ A variety of alpha‐cyano‐cinnamate derivatives were studied, the most widely used one being UK5099, with many more recently identified, as described below (Table 1). Additional classes of compounds have been shown to inhibit MPC, including several thiazolidinediones (TZDs)^82^ (Table 2), the cGMP‐specific phosphodiesterase (PDE) inhibitor zaprinast,^83^ the anti‐cancer agent lonidamine,^84^ coumarin derivatives,^27^ and quinolone antibiotics^44^ (Table 3). Although these compounds were proposed to have different primary targets and most indeed are not specific for MPC, some are high affinity binders and have provided valuable information in advancing MPC pharmacology. In this section, we discuss the chemical features of the currently known MPC inhibitors and their proposed uses as therapeutic agents.

**TABLE 1:** Cyano‐cinnamates and derivatives.

Compound name	Chemical structure	Other targets	MPC Ki/IC_50_/EC_50_	Experimental approach	References
CHC *a‐cyano‐4‐hydroxycinnamic acid*	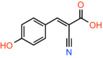	Monocarboxylate transporters	IC_50_: 1500 nM	Substrate transport in isolated mitochondria	[71]
UK5099 *(2E)‐2‐Cyano‐3‐(1‐phenyl‐1H‐indol‐3‐yl)acrylic acid*	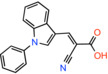	Monocarboxylate transporters	IC_50_: 50 nM	Substrate transport in isolated mitochondria	[71]
			IC_50_: 53 nM	Substrate transport in proteoliposomes	[15]
BE1976 *(E)‐3‐[5‐(1,3‐Benzothiazol‐2‐yl)‐2‐Furyl]‐2‐Cyanoacrylic Acid*	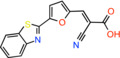	Not reported	EC_50_: 33 nM	Effect on pyruvate‐driven respiration	[60]
BE1978 *(E)‐2‐Cyano‐3‐(1‐Phenyl‐4‐Pyrazolyl) acrylic Acid*	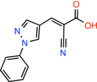	Not reported	EC_50_: 117 nM	Effect on pyruvate‐driven respiration	[60]
BE1980 *(E)‐2‐Cyano‐3‐(3,5‐Dimethyl‐1‐Phenyl‐4‐Pyrazolyl) acrylic Acid*	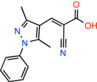	Not reported	EC_50_: 162 nM	Effect on pyruvate‐driven respiration	[60]
BE1984 *(E)‐2‐Cyano‐3‐(p‐Tolyl)acrylic Acid*	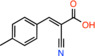	Not reported	EC_50_: 1530 nM	Effect on pyruvate‐driven respiration	[60]
BE2617 *(E)‐2‐Cyano‐3‐(4‐Phenyl‐3‐Pyrazolyl)acrylic Acid*	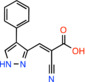	Not reported	EC_50_: 39 nM	Effect on pyruvate‐driven respiration	[60]
BE2623 *(E)‐2‐Cyano‐3‐[4‐(p‐Tolyl)‐3‐Pyrazolyl]acrylic Acid*	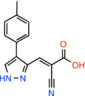	Not reported	EC_50_: 731 nM	Effect on pyruvate‐driven respiration	[60]
JXL020 *(E)‐3‐(1‐(3,5‐Bis(trifluoromethyl)benzyl)‐1H‐indol‐3‐yl)‐2‐cyanoacrylic Acid*	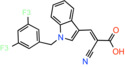	Not reported	EC_50_: 17 nM	Effect on pyruvate‐driven respiration	[88]
JXL069 *(E)‐3‐(1‐(3,5‐Bis(trifluoromethyl)benzyl)‐1H‐pyrrolo[2,3‐b]pyridin‐3‐yl)‐2‐cyanoacrylic Acid*	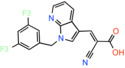	Not reported	EC_50_: 43 nM	Cell‐based lactate production/Effect on pyruvate‐driven respiration	[88]
JXL050 *(E)‐3‐(1‐(3,5‐Bis(trifluoromethyl)benzyl)‐4‐chloro‐1H‐indol‐3‐yl)‐2‐cyanoacrylic Acid*	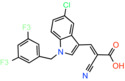	Not reported	Not reported	Cell‐based lactate production/Effects on pyruvate‐driven respiration	[88]
JXL051 *(E)‐3‐(1‐(3,5‐Bis(trifluoromethyl)benzyl)‐4‐bromo‐1H‐indol‐3‐yl)‐2‐cyanoacrylic Acid*	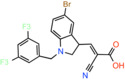	Not reported	Not reported	Cell‐based lactate production/Effects on pyruvate‐driven respiration	[88]
JXL052 *(E)‐3‐(1‐(3,5‐Bis(trifluoromethyl)benzyl)‐4‐fluoro‐1H‐indol‐3‐yl)‐2‐cyanoacrylic Acid*	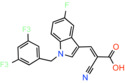	Not reported	Not reported	Cell‐based lactate production/Effects on pyruvate‐driven respiration	[88]
Compound 2 *2‐cyano‐3‐(5‐phenyl‐2‐furyl)acrylic acid*	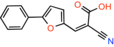	Not reported	IC_50_: 12 nM	Substrate transport in proteoliposomes	[15]
Compound 7 *2‐cyano‐3‐[5‐(2‐nitrophenyl)‐2‐furyl]acrylic acid*	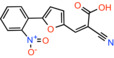	Not reported	IC_50_: 5 nM	Substrate transport in proteoliposomes	[15]

**TABLE 2:** Diones, thiazolidines, and thiazolidinediones.

Compound name	Chemical structure	Other known targets	MPC Ki/IC_50_/EC_50_	Experimental approach	References
GW604714X *(5Z)‐5‐{5‐[6‐(4‐Acetyl‐1‐piperazinyl)‐3‐nitro‐2‐pyridinyl]‐2‐fluorobenzylidene}‐1,3‐thiazolidine‐2,4‐dione*	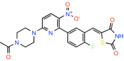	Monocarboxylate transporters, K_ATP_ channels	Ki: 0.06 nM	Effect on pyruvate‐driven respiration	[85]
GW450863X *(5Z)‐5‐[(2,5‐dimethoxyphenyl)methylidene]‐1,3‐thiazolidine‐2,4‐dione*	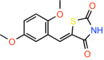	Monocarboxylate transporters, K_ATP_ channels	Ki: 0.60 nM	Effect on pyruvate‐driven respiration	[85]
Pioglitazone *5‐[[4‐[2‐(5‐ethylpyridin‐2‐yl)ethoxy]phenyl]methyl]‐1,3‐thiazolidine‐2,4‐dione*	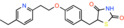	Peroxisome proliferator‐ activated receptor gamma (PPAR‐γ)	Not reported	Effect on pyruvate‐driven respiration	[82]
			Not reported	Substrate transport in proteoliposomes	[15]
Rosiglitazone *5‐[[4‐[2‐[methyl(pyridin‐2‐yl)amino]ethoxy]phenyl]methyl]‐1,3‐thiazolidine‐2,4‐dione*	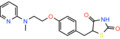	Peroxisome proliferator‐ activated receptor gamma (PPAR‐γ)	EC_50_: 9000 nM	Effect on pyruvate‐driven respiration	[82]
			Not reported	Substrate transport in proteoliposomes	[15]
MSDC‐0160 *5‐[[4‐[2‐(5‐ethylpyridin‐2‐yl)‐2‐oxoethoxy]phenyl]methyl]‐1,3‐thiazolidine‐2,4‐dione*	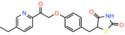	Peroxisome proliferator‐ activated receptor gamma (PPAR‐γ)	EC_50_: 5000 nM	Effect on pyruvate‐driven respiration	[82]
			IC_50_: 2700 nM	Substrate transport in proteoliposomes	[15]
Carsalam *1,3‐benzoxazine‐2,4‐dione*	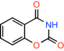	Bacterial type II topoisomerase enzymes, DNA gyrase and DNA topoisomerase IV	Not calculated	BRET/Effect on pyruvate‐driven respiration	[44]
Nitrofurantoin *1‐[(E)‐(5‐nitrofuran‐2‐yl)methylideneamino]imidazolidine‐2,4‐dione*	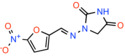	Inhibits bacterial DNA, RNA, and cell wall protein synthesis	IC_50_: 3300 nM	Substrate transport in proteoliposomes	[15]
(E)‐5‐(4‐Hydroxybenzylidene) thiazolidine‐2,4‐dione	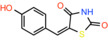	Not reported	IC_50_: 762 nM	Mitochondrial respiration in Drosophila	[97]
(E)‐5‐(3‐Hydroxy‐4‐methoxybenzylidene)thiazolidine‐2,4‐dione	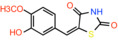	Not reported	Not reported	Mitochondrial respiration in Drosophila	[97]

**TABLE 3:** Other MPC inhibitors.

Compound name	Chemical structure	Other known targets	MPC Ki/IC_50_/EC_50_	Experimental approach	References
Zaprinast *5‐(2‐propoxyphenyl)‐2,6‐dihydrotriazolo[4,5‐d]pyrimidin‐7‐one*	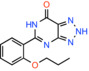	Agonist of GPR35, inhibitor of phosphodiesterase and glutaminase	Not calculated	Substrate transport in isolated mitochondria	[83]
			IC_50_: 321 nM	Substrate transport in proteoliposomes	[15]
Lonidamine *1‐[(2,4‐dichlorophenyl)methyl]indazole‐3‐carboxylic acid*	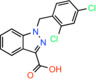	Mitochondrially‐bound hexokinase, respiratory complex II, monocarboxylate transporters	Ki: 2500 nM	Substrate transport in isolated mitochondria	[84]
			IC_50_: 4600 nM	Substrate transport in proteoliposomes	[15]
7ACC2 *7‐[benzyl(methyl)amino]‐2‐oxochromene‐3‐carboxylic acid*	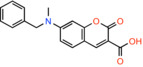	Monocarboxylate transporters	Not calculated	BRET/Effect on pyruvate‐driven respiration	[44]
7ACC1 *7‐(diethylamino)‐2‐oxochromene‐3‐carboxylic acid*	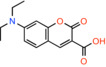	Monocarboxylate transporters	Not calculated	BRET/Effect on pyruvate‐driven respiration	[44]
Entacapone *(E)‐2‐cyano‐3‐(3,4‐dihydroxy‐5‐nitrophenyl)‐N,N‐diethylprop‐2‐enamide*	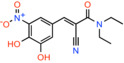	Catechol‐O‐methyl transferase (COMT)	IC_50_: 630 nM	Substrate transport in proteoliposomes	[15]
Quinolone antibiotics					
Pefloxacine *1‐ethyl‐6‐fluoro‐7‐(4‐methylpiperazin‐1‐yl)‐4‐oxoquinoline‐3‐carboxylic acid*	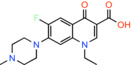	Bacterial type II topoisomerase enzymes, DNA gyrase and DNA topoisomerase IV	Not reported	BRET	[44]
Clinafloxacin *7‐(3‐aminopyrrolidin‐1‐yl)‐8‐chloro‐1‐cyclopropyl‐6‐fluoro‐4‐oxo‐1,4‐dihydroquinoline‐3‐carboxylic acid*	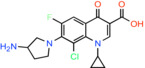	Bacterial type II topoisomerase enzymes, DNA gyrase and DNA topoisomerase IV	Not reported	BRET	[44]
Sarafloxacin *6‐fluoro‐1‐(4‐fluorophenyl)‐4‐oxo‐7‐piperazin‐1‐ylquinoline‐3‐carboxylic acid*	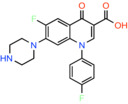	Bacterial type II topoisomerase enzymes, DNA gyrase and DNA topoisomerase IV	Not reported	BRET	[44]
Nadifloxacin *7‐fluoro‐8‐(4‐hydroxypiperidin‐1‐yl)‐12‐methyl‐4‐oxo‐1‐azatricyclo[7.3.1.0* ^*5,13*^ *]trideca‐2,5,7,9(13)‐tetraene‐3‐carboxylic acid*	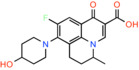	Bacterial type II topoisomerase enzymes, DNA gyrase and DNA topoisomerase IV	Not reported	BRET	[44]
Nalidixic acid *1‐ethyl‐7‐methyl‐4‐oxo‐1,8‐naphthyridine‐3‐carboxylic acid*	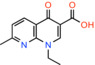	Bacterial type II topoisomerase enzymes, DNA gyrase and DNA topoisomerase IV	Not calculated	BRET/Effect on pyruvate‐driven respiration	[44]

### 7.1 Cinnamic acids, UK5099 and other derivatives

UK5099, an alpha‐cyano‐cinnamate derivative, is the most potent MPC inhibitor originally described, with potency around 50 nM, as shown in isolated mitochondria^71^ and in vitro assays with purified human MPC hetero‐dimer^15^ (Table 1). UK5099 is still considered the gold standard for pharmacological manipulation of MPC, even though cyano‐cinnamates and derivatives also inhibit the plasma membrane monocarboxylate transporters, albeit with lower potency.^85^

The mechanism of MPC inhibition by CHC or UK5099 was originally proposed to involve the formation of a covalent bond with a cysteine residing in the protein‐binding site, although the molecular identity of the MPC protein was not known at the time. This hypothesis originated from the observation that sulfhydryl reagents can inhibit pyruvate uptake in isolated mitochondria^70^ and that UK5099 and other cyano‐cinnamates happen to contain and activated double bond that could potentially react with a cysteine in the MPC binding site. Although this notion is widespread in the literature, it was never really shown that the activated double bond forms Michael addition adducts with cysteine residues in MPC. It was only shown that some of these compounds have altered absorption spectra upon addition of β‐mercaptoethanol in solution, but no evidence was provided for the interaction with an MPC cysteine.^86^ Because the presence of CHC addition product in the membrane could not be demonstrated, it was hypothesized that a covalent modification of MPC should be reversible, but direct proof for this hypothesis was never provided either.^86^ More recently, Cys54 in MPC2 was found to engage with an irreversible chemical probe,^87^ but this is chemically unrelated to the cinnamates or other MPC inhibitors.

**FIGURE 5:**
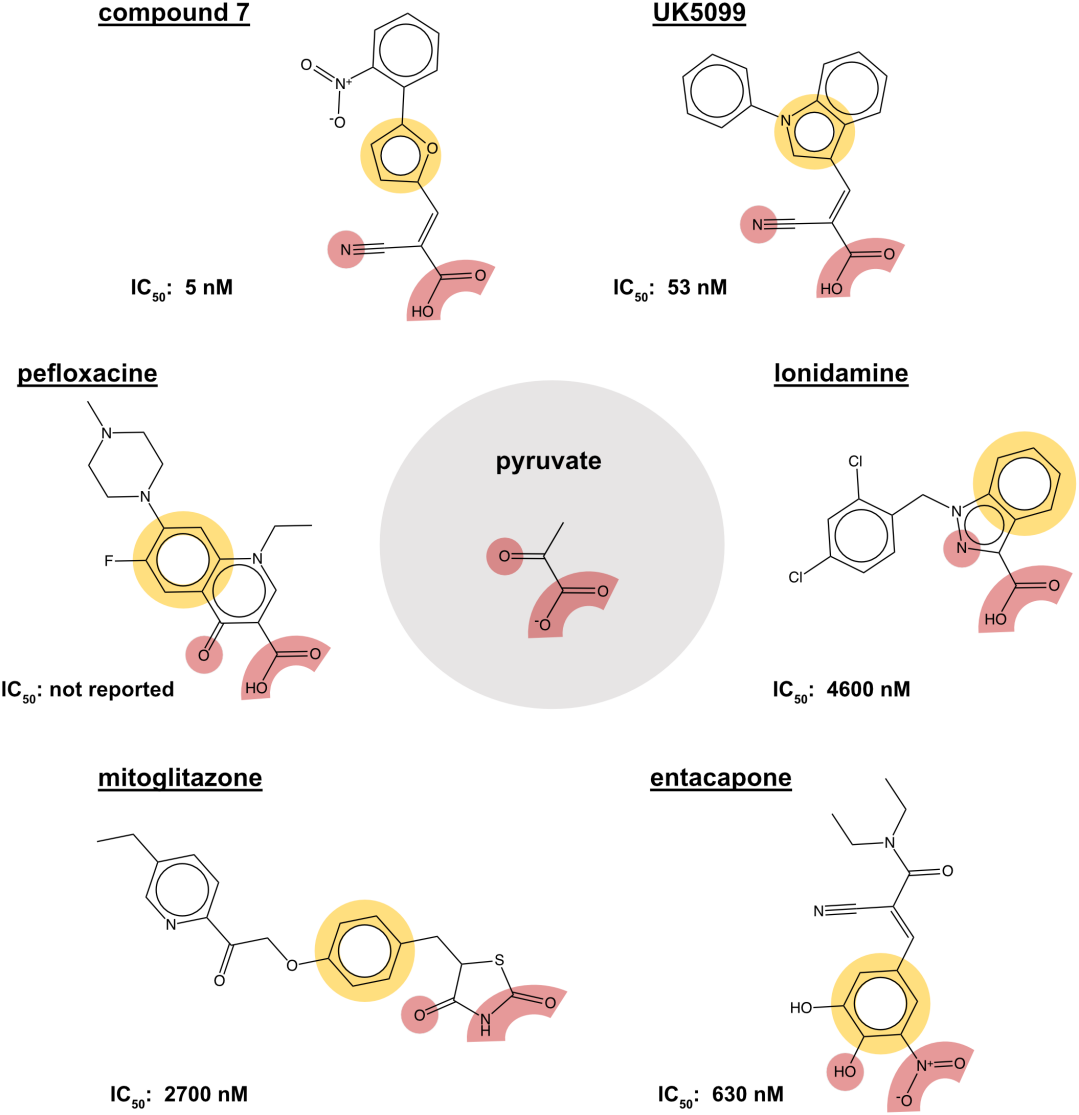
Common chemical properties of key MPC inhibitors. 2D models of pyruvate and selected MPC inhibitors are shown with key chemical properties highlighted. Polar groups mimicking pyruvate are shown in red, while hydrophobic ring features are shown in yellow spheres. IC_50_ measurements are also stated for compounds, where available.

Having purified the human MPC hetero‐dimer, we tested the above hypothesis in vitro by ESI‐MS molecular mass measurements, but importantly, no cysteine modifications were observed after binding of purified human MPC with UK5099.^15^ Furthermore, replacement of each MPC cysteine to alanine did not alter the binding of inhibitors with activated double bond. Recent computational models of MPC also place the cysteine side chains away from the dimer interface where binding of inhibitors should occur (Figure 5). Therefore, it is highly unlikely that the high potencies of UK5099 and derivatives can be explained by the formation of a covalent bond. The irreversibility observed in cellular studies can most likely be attributed to non‐covalent high affinity binding.

Still, UK5099 contains structural alerts and has generally been considered to be incompatible with therapeutic applications, mainly due to the existence of the activated double bond. It has, however, been used to treat hair loss with topical application in mice and a new set of derivatives has been developed toward that goal^88^ (Table 1). After it was shown that lactate generation is critical for the activation of hair follicle stem cells,^89^ MPC genetic or pharmacological inhibition by UK5099 was shown to be efficient in increasing lactate levels and restoring hair follicle stem and progenitor cells.^90^ Liu and colleagues synthesized and tested a series of UK5099 analogues on MCF10A cells by measuring their effect on lactate production, which is an indirect functional assay for MPC.^88^ Although a direct quantitative assay for MPC binding or inhibition was not applied, the authors proposed that when the N‐phenyl group of UK5099 was substituted by a 3,5‐bis(trifluoromethyl)benzyl group, the potency, as measured by the effect on oxygen consumption rates, increased.

Recently, our group as well as Hegazy and co‐workers have proposed a series of non‐indole MPC inhibitors that are still structurally related to alpha‐cyano‐cinnamates, as they maintain the cyanide and carboxyl groups^15^, ^60^ (Table 1). We have shown that these groups, providing essential polar interactions, are critical for high affinity binding.^15^ A single aromatic ring adjacent to this hydrogen bond acceptor trio is enough for high affinity inhibition, whereas two hydrophobic rings provide high inhibitory potency, in the low nanomolar range. Further to this, addition of a nitro group to 2‐cyano‐3‐(5‐phenyl‐2‐furyl)acrylic acid at position R1 of the second aromatic ring increases affinity by 10‐fold, providing the highest reported affinity of 5 nM for 2‐cyano‐3‐[5‐(2‐nitrophenyl)‐2‐furyl]acrylic acid^15^ (Table 1).

### 7.2 Diones, thiazolidines, and thiazolidinediones

Thiazolidinediones are insulin sensitizers used for the treatment of Type II diabetes.^91^, ^92^ However, they have been discontinued in many countries due to serious side effects including cardiovascular risk, bone loss and adiposity. They are known to exert their action via the peroxisome proliferator‐activated receptor gamma (PPARγ), which has been considered their primary target and responsible for the observed side effects. Today it is well documented that the TZDs also inhibit the MPC.

The first relevant observation for this class was that the thiazolidine compounds GW604714X and GW450863X (Table 3) inhibit pyruvate transport in isolated mitochondria with high affinities.^85^ Later, the TZDs, pioglitazone, rosiglitazone, and troglitazone were also found to inhibit pyruvate‐driven respiration in different cell types.^82^, ^93^ This observation further supported a previous hypothesis that the TZDs exert their action, at least in part, via a mitochondrial target, which could therefore be MPC. Indeed, new generation TZDs such as MSDC‐0160 (mitoglitazone) and MSDC‐0602K were developed to have decreased affinity for PPARγ and were proposed to maintain the therapeutic benefit.^94^, ^95^ MSDC‐0602 K has been considered in clinical trials for the treatment of NASH^18^, ^19^ and MSDC‐0160 for Alzheimer's disease.^96^ Additionally, a deuterium‐modified (R)‐pioglitazone which lacks in vitro activity for PPARγ is in clinical trials for the treatment of NASH.^21^ However, pioglitazone did not inhibit MPC transport in human MPC proteoliposomes to more than 50% of control even at a high concentration of 100 μM.^15^ Overall, the inhibitory potency of these compounds, especially of pioglitazone, for the MPC hetero‐dimer is low compared to other MPC inhibitors.^15^ Although lower potencies could be beneficial for certain therapeutic purposes where moderate MPC modulation is necessary, the low specificity of TZDs dictates further development.

A recent effort to identify new TZDs that are not enantiomer mixtures^97^ (Table 2) further highlighted the importance of the thiozolidine component for MPC binding. The study did not provide quantitative measurements of MPC inhibition for most of these compounds or direct comparison with the TZDs but reveals that the essential interactions are within the thiozolidine moiety, consistent with our hypothesis.^15^ Interestingly, (E)‐5‐(4‐hydroxybenzylidene) thiazolidine‐2,4‐dione appears more potent than the TZDs (Table 2). Even more, diones also bind MPC. Nitrofurantoin, a dione used as an antibiotic for urinary tract infections, binds to human MPC and inhibits pyruvate transport in proteoliposomes with a potency similar to that of the TZDs.^15^ Another dione, carsalam, a non‐steroidal anti‐inflammatory agent, was also shown to increase the BRET signal during a chemical compound screen.^44^ These findings are in agreement with the presence of pyruvate mimics within diones, thiazolidines and TZDs^15^ (Figure 5).

### 7.3 Other classes of MPC inhibitors

A much less studied inhibitor of the MPC is Zaprinast, a PDE inhibitor and lead compound in the development of sildenafil (Viagra). Zaprinast is also an agonist of GPR35^98^ and an inhibitor of glutaminase.^99^ Although not specific for MPC, it inhibits pyruvate‐driven oxygen consumption in brain mitochondria and pyruvate uptake in isolated mitochondria.^44^, ^83^ In in vitro studies using purified human MPC in proteoliposomes we have shown that zaprinast has high inhibitory potency (IC_50_: 321 nM), ranking above the TZDs^15^ (Table 3). Overall, Zaprinast is a better inhibitor for the MPC than it is for PDEs, inhibition of which requires 50–200 μM.^83^

Lonidamine is a derivative of indazole‐3‐carboxylic acid and has been known to inhibit aerobic glycolysis and energy metabolism selectively in tumor cells. It is currently used in clinical practice as a combination therapy in cancer treatment to sensitize tumors in radiotherapy and chemotherapy.^100^ Its action on MPC is now well established, as it can inhibit pyruvate uptake in isolated mitochondria^84^, ^101^ and the human MPC hetero‐dimer reconstituted into liposomes.^15^ Its inhibitory potency in the low micromolar range is comparable to that of the TZDs^15^ (Table 3). Lonidamine has at least three more known targets, the mitochondrially bound hexokinase, the respiratory complex II^101^, ^102^ and the monocarboxylate transporters.^101^ However, it has higher affinity for MPC than for the other proposed targets^101^ and it is compelling to hypothesize that its therapeutic action via energy metabolism control of cancer cells is also related to MPC inhibition, or at least in part.

MPC is also inhibited by the monocarboxylate transporter inhibitors 7ACC2^14^, ^27^, ^44^ and 7ACC1, which are coumarin derivatives. 7ACC2 inhibits both the yeast Mpc1/Mpc3 with low potency^14^ and the human complex,^27^, ^44^ although the inhibitory potency is not known in this case. MPC inhibition by 7ACC2 induces cytotoxic effects in cells together with glycolysis stimulation and inhibition of mitochondrial respiration while tumor xenografts are sensitized to radiotherapy by 7ACC2.^27^

A series of quinolone antibiotics have also been proposed as MPC binders.^44^ Specifically, pefloxacine, clinafloxacin, nalidixic acid, sarafloxacin, nadifloxacin, and moxifloxacin (Table 3), increased the BRET signal, suggesting interaction with the MPC. When tested for their ability to inhibit pyruvate driven respiration, nalidixic acid was more potent than other quinolones, but much less potent than 7ACC2. Binding affinities and inhibition of pyruvate transport were not demonstrated.

Lastly, entacapone, used in combination therapy for Parkinson's disease, inhibits transport of human MPC in vitro with high potency, at 630 nM.^15^

### 7.4 Knowns and unknowns of the binding mechanism

The substrate and inhibitor binding mechanisms of the MPC are currently unknown due to the absence of structural information, but also because assays, which are needed for structure–function studies, were only recently developed.^14^, ^15^ On the other hand, the MPC pharmacology is quite extended and allows for useful observations and testable hypotheses.

Insights into the location and characteristics of the inhibitor binding site can be obtained by biochemical and biophysical studies on the MPC protein. First, the binding site must be forming in the interface between the two MPC protomers and potentially overlaps with the binding site of pyruvate. In support of the latter, small molecule MPC inhibitors share common features with pyruvate. Specifically, they all contain an arrangement of three polar groups^15^ which mimic the carboxyl and ketone groups of pyruvate^15^ (Figure 5). For example, in UK5099 and related molecules, this requirement is satisfied by the carboxyl and the nitrile group, respectively. In lonidamine, the carboxyl group and N2 nitrogen of the indazole ring and in pefloxacine the carboxyl and ketone group of the quinoline ring, respectively, are potential substrate mimics. In diones and TZDs, the dione oxygens and the N3 nitrogen of the thiazolidine ring and in entacapone the nitro group and adjacent hydroxyl group of the benzyl ring could also provide groups mimicking pyruvate (Figure 5). The substrate and inhibitors differ by the presence of one or more hydrophobic moieties, which in turn contribute to the inhibitory potency through the size and number of additional groups. In support of the binding site forming in the hetero‐dimer interface, both human hetero‐dimers bind known MPC inhibitors with high affinities,^15^ whereas the individual protomers do not bind any of the ligands with the expected affinity or at all.^55^, ^61^

The longstanding theory that binding of cyano‐cinnamates and derivatives is mediated by a reversible covalent bond with an MPC cysteine has been disputed by in vitro mass spectrometry data and alanine scanning mutagenesis on purified MPC hetero‐dimers.^15^ Similar mutagenesis approaches are revealing residues that are important for MPC function. Three different amino acid residues have been proposed so far to be part of the human MPC binding pocket, Phe66 in MPC1 and Asn100 with Lys49 in MPC2.^60^ Structural analysis of the liganded complex or full mapping of the putative binding pocket via mutagenesis and binding assays is required to elucidate the binding mechanism and to facilitate drug discovery efforts.

## 8 ACKNOWLEDGMENTS

This work was supported by the Medical Research Council (Grant number MC_UU_00028/2 to E.R.S. Kunji)

## 9 CONCLUDING REMARKS

Increasing evidence places the MPC among the promising targets for major metabolic diseases, such as the non‐alcoholic fatty liver disease for which there is currently no approved pharmacological treatment. Therefore, there is a pressing need to develop specific MPC inhibitors to facilitate studies on the physiological role of MPC in humans, but also to develop new treatments. MPC has interesting and extended pharmacology, but so far, most inhibitors have already known primary or alternative targets, which is naturally highly problematic. Understanding the structure and function of the protein in atomic detail will facilitate the development of potent and specific inhibitor compounds. Additionally, the field will have to work further towards understanding the consequences of MPC inhibition in different tissues and to develop tissue‐specific strategies for MPC targeting.

## CONFLICT OF INTEREST STATEMENT

The authors declare no conflict of interest.
